# Patterns of psychiatric hospitalizations of Arab and Jewish adults with chronic psychotic disorders, before and after national mental health reforms, Israel, 1991–2016

**DOI:** 10.1186/s12888-023-05132-w

**Published:** 2023-09-05

**Authors:** Sharon Florentin, Paola Rosca, Inbal Reuveni, Razek Haled, Yehuda Neumark

**Affiliations:** 1 Department of Psychiatry, Hadassah Medical Center, Jerusalem, Israel; 2 Faculty of Medicine, The Hebrew University of Jerusalem, Jerusalem, Israel; 3 Department for the Treatment of Substance Abuse, Ministry of Health; The Hebrew University of Jerusalem, Jerusalem, Israel; 4 Braun School of Public Health & Community Medicine, Faculty of Medicine, The Hebrew University of Jerusalem, Jerusalem, Israel

**Keywords:** Schizophrenia, Chronic psychotic disorders, Psychiatric hospitalization, Arabs, Jews, Ethnic minority, Sex differences, National psychiatric registry

## Abstract

**Background:**

Members of the Arab minority in Israel are at increased risk of developing mental illness, although less likely to seek care and have accessible treatment. This study compares trends in psychiatric hospitalizations between Arabs and Jews with chronic psychotic disorders, before and after introduction of the Community Rehabilitation of Persons with Mental Disability Law in 2000, and governmental recognition of the need to allocate resources for patients with co-occurring substance use disorder and mental illness in 2010.

**Methods:**

The National Psychiatric Case Registry provided data on 18,684 adults with schizophrenia/schizoaffective disorder, hospitalized in 1991–2016 (at least once in 2010–2015). Repeated-measures ANOVA was used to measure the effect (and interactions) of population-group (Arabs and Jews), time-period (Period_1_: 1991–2000, Period_2_: 2001–2009, Period_3_: 2010–2016) and sex, on average length of stay (LOS), annual number of hospitalizations and hospitalization days.

**Results:**

The proportion of Arab patients hospitalized with a diagnosis of chronic psychotic disorder (14.4%) was significantly lower than their proportion in the general population (21%), and their average age at first hospitalization (28.4 years) was older than that of Jewish inpatients (27.0 years). The average number of hospitalization days and LOS of Jewish patients were double that of Arab patients in Period_1_. Following implementation of the Rehabilitation Law, hospitalization days increased among Arab patients and decreased slightly among Jewish patients, such that by Period_3_ the average number of hospitalization days was similar among Jewish (41) and Arab (37) patients. The increase in hospitalization days among Arab patients was limited to men with no change noted among women. The number of hospitalization days among Arab women was about half that of Jewish women (p < 0.0001).

**Conclusions:**

The findings reveal a narrowing of disparities in psychiatric hospitalizations between Arab and Jewish patients in Israel over time. However, among Arab women the number of hospitalization days remains considerably lower than that of Jewish women, raising concerns that Arab women may be receiving insufficient care. Further study is needed to fully understand the underpinnings of these disparities, although increasing the number of Arabic-language mental health services and providing psycho-education, will help further close the gap.

## Background

Ethnic minorities and immigrants are at increased risk of developing mental illness including psychotic disorders [1–5]. Underlying this increased risk is a complex set of social factors that include socio-economic disadvantage, as well as discrimination and alienation [6–9]. Despite the higher incidence of psychiatric disorders among minority groups, in many countries, they are less likely to seek and to have access to care [10–13] and have a higher probability of involuntary hospitalization [14–17]. Mental health treatment gaps exist also in Israel between the Jewish majority and the Arab minority - who constitute 21% of the country’s population (of whom 83% are Muslim, 9% are Druze and 8% are Christians). The Arab minority in Israel, while having achieved a remarkable improvement in health and life expectancy, still suffers from social disparity compared with the Jewish population. The Arab population has an increased risk of emotional distress which may be related to social stressors, psychosocial disempowerment and self-appraisal of lower social status [18, 19]. They are also less likely to seek mental health care [18–20] and the delay between first diagnosis and commencement of treatment is greater among Arab patients [19]. The limited availability and accessibility of linguistically and culturally appropriate care, hampers treatment seeking [21].

Lurie and colleagues [22] found that among people hospitalized in a psychiatric ward in Israel between 2003 and 2013, the mean length of stay per admission was about half as long among Muslim-Arab patients compared with Jewish and Christian-Arab patients. The mean number of days between hospitalizations was also lower among Muslims, resulting in a slightly higher number of hospitalizations. Court-ordered involuntary hospitalizations were twice as common among Muslim-Arab patients (16.3%) as Jewish patients (7.3%). Over the years, the gap in rate of hospitalizations between Jewish and Arab patients diminished [22].

A mental health reform was implemented in Israel in 2000 that led to the opening of community rehabilitation services for people with severe mental illness, alongside a reduction in the number of psychiatric hospital beds [23]. A decade later, in 2010, the Israeli parliament officially recognized the need to allocate additional resources for patients with co-occurring substance use disorder and mental illness, or dual-diagnosis (DD) patients [24]. Adoption of these policies catalyzed a gradual process of opening hospital-based and community-based services tailored for DD patients. Use of rehabilitation services is associated with fewer hospital days among people with severe mental illness [25], particularly those with chronic psychotic disorders with or without DD [26, 27].

### Study Aim

In this study we examined, for the first time, hospitalization patterns [frequency of hospitalizations, mean length of stay (LOS) and annual hospital days per person] among Arab and Jewish patients in three time periods: 1991–2000, 2001–2009, and 2010–2016) - before and after implementation of the mental health reforms. Specifically, we tested three a-priori hypotheses: (1) the frequency of hospitalizations is greater and LOS shorter among Arab patients; (2) in both Arab and Jewish patients, LOS is shorter among women; (3) Arab-Jewish differences in hospitalization characteristics have converged over time.

## Methods

Data for this study was extracted from the Israel’s National Psychiatric Case Registry (NPCR) of the Ministry of Health. The NPCR is the official registry of all psychiatric admissions and discharges countrywide since 1950 [29]. The data included 18,684 patients aged 18–65 years hospitalized in a psychiatric ward during the period 1963–2016, with an ICD-10 diagnosis of schizophrenia (F20) or schizoaffective disorder (F25) at their last discharge, and with at least one hospitalization occurring during the years 2010–2015. Of the 18,684 patients, 15,145 self-identified as Jewish and 2,556 self-identified as Arabs. As previously described [26–28], we restricted the sample to those persons hospitalized at least once since 2010 in order to ensure that the data and the findings are relevant and timely.

For each hospitalization, a Substance Use Disorder (SUD) diagnosis was made based on a recorded ICD-10 diagnosis of F10-F19 (excluding F17, nicotine dependence) in the categories of dependence and harmful use (DSM diagnoses are not used in Israel) and/or a psychiatrist-documented indication of alcohol and/or substance abuse. We adopted a conservative approach, and classified patients as DD only if they met SUD criteria on at least two hospitalizations, or at least 20% of their hospitalizations, rather than the more common ‘lifetime’ or first-hospitalization criteria. Each individual’s hospitalization history was documented from the first hospitalization until the end of 2016. Twenty-nine patients with anomalous numbers of hospitalizations (≥80) were excluded from the study. A total of 18,684 patients with 168,377 hospitalizations were included in the analysis [26–28].

For each patient, the following hospitalization measures were calculated: average length of stay (LOS), annual number of hospitalizations, and annual number of hospitalization days. For a complete case analysis, repeated-measures ANOVA (with Greenhouse-Geisser correction) was used to measure associations with population group (Arabs, Jews), time-period (Period_1_: 1991–2000, Period_2_: 2001–2009, Period_3_: 2010–2016) and gender, as well as interactions between these variables on hospitalization measures. By way of a sensitivity analysis, linear mixed effects models with random intercept at the participant level adjusted for gender and ethnicity were also run to compare all individuals across the periods. The data were analyzed using IBM® SPSS® Statistics, Version 24.0 and STATA 16.

The study was conducted in accordance with the Declaration of Helsinki and was approved by the Institutional Review Board of the Israel Ministry of Health. Data was anonymized prior to being released to the researchers.

## Results

As seen in Table 1, Jewish patients were, on average, 2.4 years older than Arab patients and younger at first hospitalization. The proportion of females was higher in the former group. Number of hospitalizations per patient and LOS were higher among Jewish patients. A higher proportion of Jewish patients (75%) than Arab patients (70%) had ever been hospitalized involuntarily.

**Table 1:** Demographic and hospitalization characteristics of Israeli Arab and Jewish patients (aged 18–65) with a psychiatric hospitalization during the period 1963–2015, among persons hospitalized in the period 2010–2015

Characteristic	Arab patients (N = 2556)	Jewish patients (N = 15145)	p-value
% Female	27.6	35.3	< 0.0001**
% with Substance Use Disorder	28.1	29.7	0.099**
Mean age at first hospitalization (SD)	28.4 (9.1)	27.0 (9.9)	< 0.0001*
Mean number of hospitalizations per patient (SD)	7.7 (8.9)	9.33 (10.2)	< 0.0001*
Mean length of stay (days)	62.4 (133.7)	101.5 (242.8)	< 0.0001*
% of patients ever hospitalized involuntarily			< 0.0001**
Psychiatrist order	62.5	69.6	
Court order	27.3	23.3	
Psychiatrist and/or Court order	70.5	74.8	

*p-value for independent t test statistic; **p value for χ^2^ statistic; ***p value for F statistic

### Annual number of psychiatric hospitalizations per patient

**Fig. 1:**
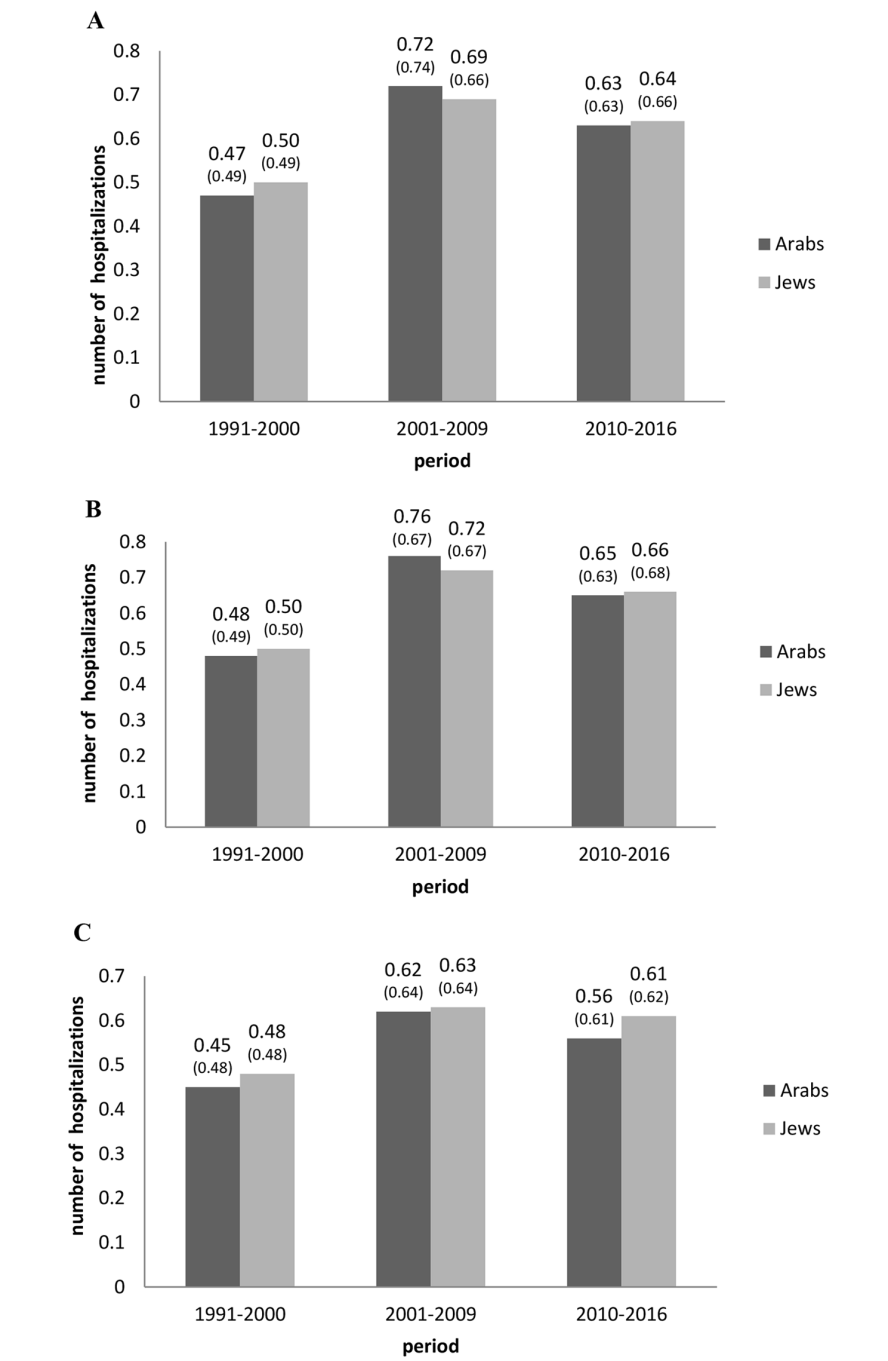
Average annual number (SE) of hospitalizations/person among Arab and Jewish patients (total, males, females), in three time periods, Israel. Note: p < 0.0001 for all comparisons

As presented in Fig. 1, broadly similar temporal trends in the average number of hospitalizations per year were noted among Arab patients (N = 544) and Jewish patients (N = 4524) who were hospitalized in all three study periods. In both groups, a marked increase in hospitalizations was seen in Period_2_ followed by a moderate decrease in Period_3_ (p < 0.001). The increase in the annual number of hospitalizations from Period_1_ to Period_2_ was steeper among Arab patients (from 0.47 to 0.72) than Jewish patients (from 0.50 to 0.69), as was the decrease from Period_2_ to Period_3_ (Fig. 1). The annual number of hospitalizations was similar among Arab and Jewish patients in all three periods, and in both groups, was lower among women than men in all three study periods (p < 0.001).

### Length of stay

**Fig. 2:**
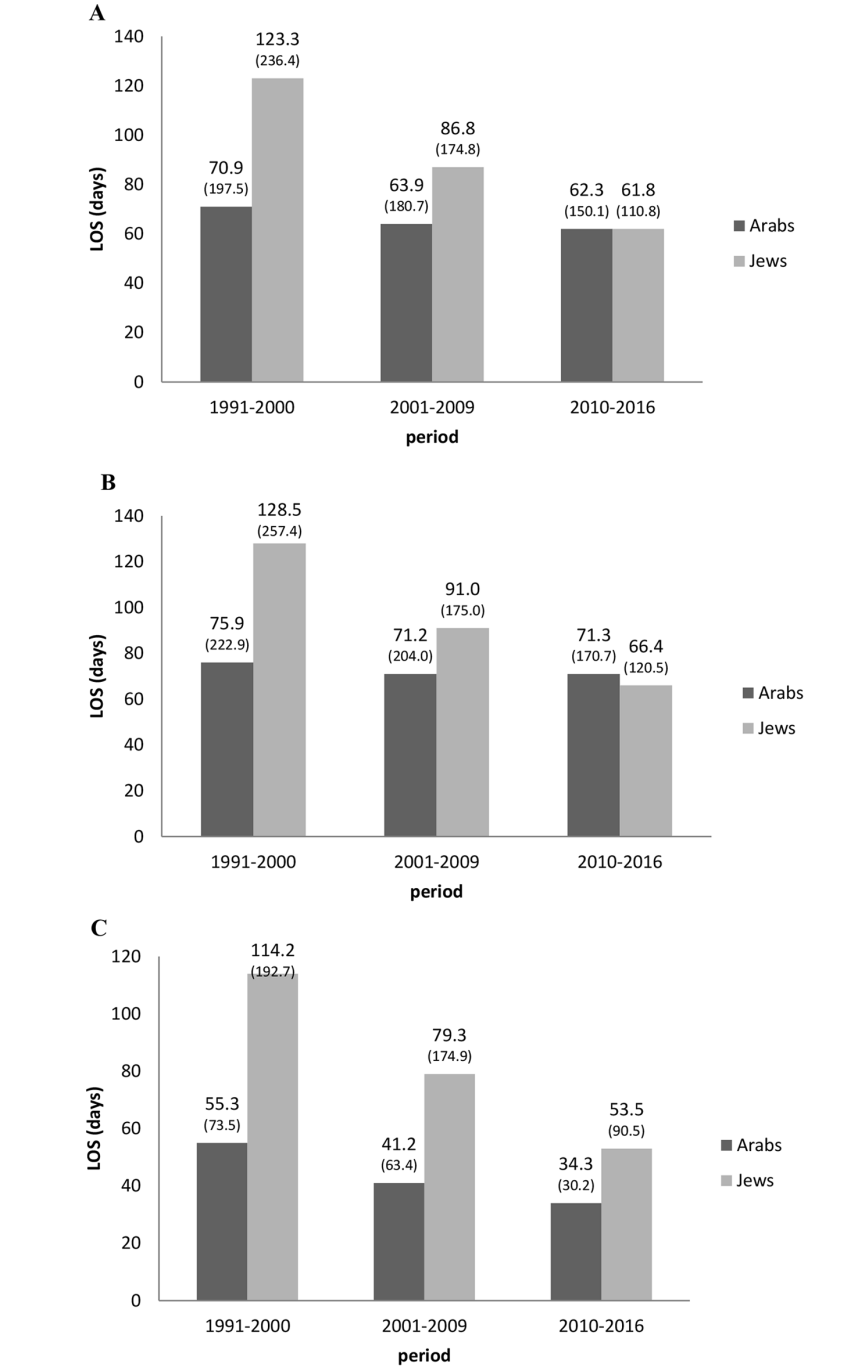
Average length of hospital stay among Arab and Jewish patients (total, males, females), in three time periods, Israel. Note: p < 0.0001 for all comparisons

In Period_1_, the mean LOS among Jewish patients (N = 4461) was nearly two-fold longer than among Arab patients (N = 541) − 123.3 days and 70.9 days, respectively (p < 0.0001) (Fig. 2). LOS remained longer among Jewish patients in Period_2_ despite a marked decrease in the number of hospitalizations among Jewish patients from Period_1_ (123.4) to Period_2_ (86.8) (p < 0.0001) and a modest decrease among Arab patients - from 70.9 to 63.9 (p < 0.0001). This gap narrowed further with time, and in Period_3_ the average LOS was nearly identical among Jewish patients (61.8 days) and Arab (62.3 days) patients. The LOS of women was significantly shorter than that of men in all three periods, with a greater gap between Arab women and men than among Jewish women and men (p < 0.0001). LOS decreased between the three periods similarly among Jewish women and men. A decrease in LOS was also noted among Arab women across all three periods, while among Arab men there was a moderate decrease in LOS between Period_1_ and Period_2_ and no change between Period_2_ and Period_3_ (p < 0.0001 for period*population-group*sex interaction).

### Annual days of psychiatric hospitalization per patient

**Fig. 3:**
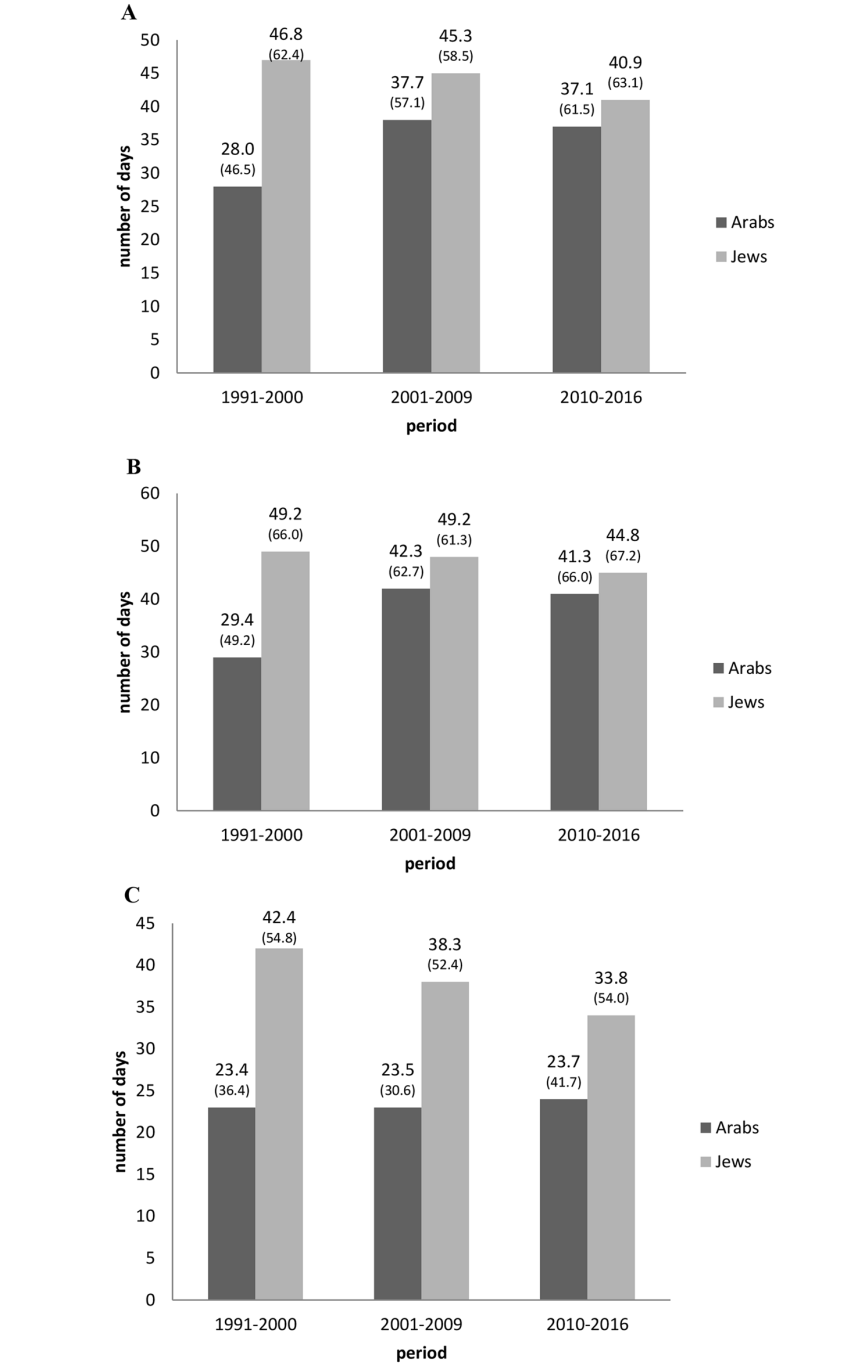
Average annual number of hospital days/person among Arab patients and Jewish patients (total, males, females), in three time periods, Israel. Note: p < 0.0001 for all comparisons

As seen in Fig. 3, in Period_1_, the average annual number hospitalization days among Jewish patients (46.8) was almost twice that of Arab patients (28.0) (p < 0.0001). Among Arabic patients, the number of hospitalization days increased to 37.7 in Period_2_, while remaining virtually unchanged among Jewish patients. The difference in the number of hospitalization days between Jewish and Arabic patients narrowed further in Period_3_, as the number of days remained constant among Arab patients and decreased slightly among Jewish patients from Period_2_ to Period_3_ (p < 0.0001 for period*population-group interaction).

As with LOS, the annual number of hospitalization days was significantly lower among women than men in all three periods, with a greater gap between Arab women and men than among Jewish women and men. Among Jewish men and women there was a decrease in hospitalization days over the periods, which was more pronounced among women. However, among Arab men there was a substantial increase in hospitalization days between Period_1_ and Period_2_, while among women there was no noticeable change in the number of hospitalization days over time (p < 0.0001 for period*population-group*sex interaction).

The data were reanalyzed using linear regression modelling that allowed for inclusion of all persons hospitalized in any of the three time periods. Significant ethnicity- and gender-adjusted differences (p < 0.0001) across the time periods were noted in all three hospital indicators. These differences were similar to those obtained in the repeated measures models presented above, which included only those persons hospitalized in all three periods. The annual number of hospitalizations in Period_2_ and in Period_3_ was 0.1 higher than in Period_1_ (0.41; SE 0.01). LOS was 52.2 days lower in Period_2_ than in Period_1_ (139.3 days; SE 2.2) and 87.1 days lower in Period3 than in Period_1_. The annual number of hospitalization days in Period_1_ was 42.6 (SE 0.62) and decreased by 9.1 days in Period_2_ and by 12.5 days in Period_3_. Significant effects for ethnicity and sex were also found. Among Arab patients, LOS was shorter by 24.1 days (SE 3.2) than among Jewish patients, and Arab patients experienced 9.4 days (SE 0.9) fewer hospitalization days, on average.

## Discussion

Schizophrenia is estimated to develop in about 0.5% of adults, with little variation across countries [30, 31]. The Israeli Arab minority constitutes 21% of the country’s population [32], yet the proportion of Arab patients among persons hospitalized with a diagnosis of schizophrenia or schizoaffective disorder in Israel was 14.4%. Assuming there is no major underlying difference in prevalence between the two population groups, it would seem that a greater proportion of Arabs with psychotic disorder in need of hospital care, are not hospitalized.

As described above, and in keeping with our hypothesis, the large differences in psychiatric hospitalization patterns between Jewish and Arab patients that were noted in earlier years, narrowed over time. The most noticeable gap was in the pre-reform study period (1991–2000) when LOS and annual days of hospitalization were nearly two-fold higher among Jewish than Arab patients. A dramatic decrease in hospitalization days among Jewish patients, and a modest decrease among Arab patients, followed enactment of the Community Rehabilitation of Persons with Mental Disability Law in 2000. The law brought about a reduction of hospitalization beds and the opening of community-based rehabilitation services, primarily though, for the Hebrew-speaking (Jewish) population. Till today, there is a substantial shortage of rehabilitation frameworks for the Arab-minority. In Jerusalem, for example, the ratio of Hebrew-language rehabilitation services to Arabic-language service is over 20:1, although Arabs comprise one-third of the city’s population. In addition, only one residential detoxification facility and few community-based SUD services are available for the Arabic-speaking population [33]. Arabs comprise nearly one-quarter of the population of Israel, yet less than 2% of psychiatrists in the country are [34]. These are especially critical service gaps for young Arab adults who require a culturally appropriate therapeutic approach and who have difficulty adjusting to treatment settings geared for the Jewish population which pose linguistic and cultural challenges that likely impair patient-therapist communication.

Consistent with the tendency in the minority Arab population to avoid hospitalization and thus postpone initial admission [18–20], Arab patients are, on average, a year and a half older than Jewish patients at the time of their first hospitalization. Several factors, beyond the limited number of treatment settings and programs, may contribute to hesitancy to seek mental health care. Knowledge and awareness about mental disorders generally lags behind that for physical diseases [35], particularly among socially disadvantaged and marginalized groups. Furthermore, social stigma about mental disorders is dominant in the traditional Arabic society [36–38], especially among women, as it is in the ultra-Orthodox Jewish community [39, 40].

It is also possible that greater family support in the Arab population of Israel, and an extended family living arrangement helps prevent exacerbations that require hospitalization [41, 42]. Social and familial support may also explain the shorter length of hospital stay among Arab patients, as there may be fewer cases in which social intervention for housing is required, such as searching for an alternative post-discharge place of residence, which prolongs the hospital stay. A greater tolerance for aberrant psychotic behavior, reliance on traditional healers, and a mistrust of the public hospital system that is regarded as “westernizing medicine” by some members of the Arabic community, may also play a role [20–22].

Women experienced less frequent hospitalizations and fewer hospital days than men, as has been previously reported [22]. Although various studies did not find significant differences in LOS between men and women [43, 44], in our study women’s LOS was shorter. The average LOS in Israel is higher than in the US and other Western countries [43–45], and it is therefore possible that in longer hospitalizations, as in Israel, the better prognosis of women is also expressed in a shorter LOS compared to that of in men [31, 46]. This gender difference was more pronounced among Arab patients, perhaps due to greater pressure for early discharge to bring the woman back home. Firstly, maintaining religious practices and preserving modesty in hospital settings is a challenge among Muslim women, about which much has been written [48–52]. Also, although the Israeli Arab society is transitioning toward modernization, women are still bound by traditional cultural norms that emphasize the woman’s responsibility for “internal” tasks within the home []. Among women, the onset age of schizophrenia is typically in the mid-twenties, on average 3–5 years later than in men [31, 53, 54] (with a second peak after age 40 [31, 55]). At these ages, Arab women are more likely than Jewish women to be married and have children. In 2010, 77% of Arab women aged 25 − 20 were married compared with 51% of Jewish women, and in 2020 these percentages were 70% and 47%, respectively [Central Bureau of Statistics of Israel, 2023]. Being married is itself associated with a better prognosis and fewer hospitalizations [31]. Historically, Muslim women had much higher fertility rates than Jewish women, although the gap declined from a three-fold difference in the 1960’s to a two-fold difference even as recently as the early 2000’s and has all but disappeared in recent years [Central Bureau of Statistics of Israel, 2023]. Notwithstanding, Arab women still suffer from greater social disadvantage compared to men, compounded by their subordinate role in a patriarchal society, thus affecting their contact with mental health services [18, 56, 57].

The dramatic decrease in hospitalization days seen among Jewish patients from Period_1_ to Period_2_ is likely due to the opening of rehabilitation services in the community and the reduction of hospitalization beds. Among Arab patients, only a modest decrease in hospitalization days was noted, perhaps related to the ongoing limited availability of suitable community-based services. It is plausible that members of the Arab population may have reservations about seeking rehabilitation, similar to their hesitancy towards hospitalization. The decrease in hospital days coincided with an increase in the number of hospitalizations in both groups. It is possible that among the Jewish patients the increase in the number of hospitalizations is related to the shortening of LOS among patients who were released before reaching full remission due to the reforms described, when the rehabilitation system was still in its infancy. Among Arab patients, the shortening of LOS is relatively minor and therefore unlikely to be the primary cause of the significant increase in hospitalizations. The increasing use of drugs (e.g., synthetic cannabinoids and cannabis) in both groups may have also contributed to the increase in the number of hospitalizations in Period_2_ [27].

Strengths and Limitations.

This study utilized data from the Israel Mental Rehabilitation Register and the National Psychiatric Case Register that captures virtually all psychiatric hospitalizations. Complete hospitalization histories were obtained for all in-patients diagnosed with schizophrenia or schizoaffective disorder in Israel in the period 2010–2015. However, the retrolective design of the study precluded the analysis of some important demographic and clinical variables unavailable in the national register, such as socioeconomic status, severity of the psychotic disorder, and level of functioning. Restricting the analyses to persons hospitalized between 2010 and 2015, rather than including all patients with the relevant diagnoses who were hospitalized during the entire study period (from 1991), limited our ability to assess whether differences in hospitalization characteristics between Arab and Jewish patients have converged over time. In addition, Twenty-nine individuals with more than 80 hospitalizations were not included in the analyses. The decision to exclude them stemmed from an impression that the excessive numbers of hospitalizations might have been due to double reporting, however they could also represent extreme cases of revolving-door patients. Regrettably, the data for these individuals is unavailable and we are unable to rerun the analyses with them included to assess the impact of the exclusion. We believe the impact is negligible given the small number excluded relative to the size of the study population.

## Conclusions

The findings of our study reveal a narrowing of disparities in psychiatric hospitalizations between Arab and Jewish patients in Israel over time. However, the number of days that Arab women spend in hospital remains considerably lower than Jewish women. This raises concerns that Arab women, who might suffer from double discrimination due to their gender and belonging to an ethnic minority, may be receiving insufficient care. The reasons for reduced help-seeking of mental health services among the Arab population are multiple and include social barriers related to being a minority group undergoing major social changes, cultural, religious and political factors, and the prevailing view that mental health and psychiatry are a westernizing issue. Increasing the number of Arabic-speaking therapists and providing psycho-education and mental health services that are tailored to Arab culture, are paramount to making treatment more accessible to the Arab community of Israel.

## Data Availability

The datasets analyzed in this study were obtained from the Ministry of Health of Israel. Access to the data is highly restricted due to the sensitive nature of psychiatric patient data. Requests to access these datasets should be directed to Mr. Reuven Eliyahu (reuven.eliahu@moh.gov.il) Data Security Office, Ministry of Health.

## Abbreviations

LOS: Length of stay
SUD: Substance use disorder

## References

[1] Tarricone I, D’Andrea G, Jongsma HE, et al. Migration history and risk of psychosis: results from the multinational EU-GEI study. Psychol Med. 2021;1–13. 10.1017/S003329172000495X. 10.1017/S003329172000495X PMC9693676 33563347

[2] Coid JW, Kirkbride JB, Barker D et al. Raised incidence rates of all psychoses among migrant groups: findings from the East London first episode psychosis study [published correction appears in Arch Gen Psychiatry. 2009;66(2):161]. Arch Gen Psychiatry. 2008;65(11):1250-8. 10.1001/archpsyc.65.11.1250. 10.1001/archpsyc.65.11.1250 18981336

[3] Morgan C, Knowles G, Hutchinson G (2019). Migration, ethnicity and psychoses: evidence, models and future directions. World Psychiatry.

[4] Jongsma HE, Karlsen S, Kirkbride JB, Jones PB (2021). Understanding the excess psychosis risk in ethnic minorities: the impact of structure and identity. Soc Psychiatry Psychiatr Epidemiol.

[5] Collazos F, Malagón-Amor Á, Falgas-Bague I (2021). Treating immigrant patients in psychiatric emergency rooms. Transcult Psychiatry.

[6] Jongsma HE, Gayer-Anderson C, Tarricone I (2021). Social disadvantage, linguistic distance, ethnic minority status and first-episode psychosis: results from the EU-GEI case-control study. Psychol Med.

[7] Montemitro C, D’Andrea G, Cesa F (2021). Language proficiency and mental disorders among migrants: a systematic review. Eur Psychiatry.

[8] Selten JP, van der Ven E, Termorshuizen F (2020). Migration and psychosis: a meta-analysis of incidence studies. Psychol Med.

[9] Lo CC, Cheng TC (2018). Social Status, discrimination, and minority individuals’ Mental Health: a secondary analysis of US national surveys. J Racial Ethn Health Disparities.

[10] Maura J, Weisman de Mamani A (2017). Mental health disparities, treatment engagement, and attrition among racial/ethnic minorities with severe mental illness: a review. J Clin Psychol Med Settings.

[11] Memon A, Taylor K, Mohebati LM (2016). Perceived barriers to accessing mental health services among black and minority ethnic (BME) communities: a qualitative study in Southeast England. BMJ Open.

[12] Alegría M, Canino G, Ríos R (2002). Inequalities in use of specialty mental health services among Latinos, African Americans, and non-latino whites. Psychiatr Serv.

[13] Kim G, Dautovich N, Ford KL (2017). Geographic variation in mental health care disparities among racially/ethnically diverse adults with psychiatric disorders. Soc Psychiatry Psychiatr Epidemiol.

[14] Davies S, Thornicroft G, Leese M, Higgingbotham A, Phelan M (1996). Ethnic differences in risk of compulsory psychiatric admission among representative cases of psychosis in London. BMJ.

[15] Terhune J, Dykxhoorn J, Mackay E, Hollander AC, Kirkbride JB, Dalman C (2022). Migrant status and risk of compulsory admission at first diagnosis of psychotic disorder: a population-based cohort study in Sweden. Psychol Med.

[16] Rodrigues R, MacDougall AG, Zou G (2019). Risk of involuntary admission among first-generation ethnic minority groups with early psychosis: a retrospective cohort study using health administrative data. Epidemiol Psychiatr Sci.

[17] Barnett P, Mackay E, Matthews H (2019). Ethnic variations in compulsory detention under the Mental Health Act: a systematic review and meta-analysis of international data. Lancet Psychiatry.

[18] Levav I, Al-Krenawi A, Ifrah A et al. Common mental disorders among Arab-Israelis: findings from the Israel National Health Survey [published correction appears in Isr J Psychiatry Relat Sci. 2008;45(2):120]. Isr J Psychiatry Relat Sci. 2007;44:104 – 13. 18080647

[19] Ponizovsky AM, Geraisy N, Shoshan E, Kremer I, Smetannikov E (2007). Treatment lag on the way to the mental health clinic among arab- and jewish-israeli patients. Isr J Psychiatry Relat Sci.

[20] Daeem R, Mansbach-Kleinfeld I, Farbstein I (2019). Barriers to help-seeking in israeli arab minority adolescents with mental health problems: results from the Galilee study. Isr J Health Policy Res.

[21] Al-Krenawi A, Levav I (2009). The epidemiology of mental health disorders among Arabs in Israel. Psychiatric and behavioral Disorders in Israel: from epidemiology to Mental Health Action.

[22] Lurie I, Fleischman A (2018). Psychiatric hospitalizations among the arab population in Israel: a historic cohort study. Isr J Psychiatry Relat Sci.

[23] Aviram U, Ginath Y, Roe D (2012). Mental health reforms in Europe: Israel’s rehabilitation in the community of persons with mental disabilities law: challenges and opportunities. Psychiatric Serv.

[24] Natan G. Treatment of Drug Dependent Persons With Co-morbid Mental Illness. Report to the Israeli Parliament (Knesset), Jerusalem (2010). Available online at: https://fs.knesset.gov.il/globaldocs/MMM/ed566b58-e9f7-e41180c8-00155d010977/2_ed566b58-e9f7-e411-80c8-00155d010977_11_10107.pdf.

[25] Hornik-Lurie T, Zilber N, Lerner Y (2012). Trends in the use of rehabilitation services in the community by people with mental disabilities in Israel; the factors involved. Isr J Health Policy Res.

[26] Florentin S, Rosca P, Bdolah-Abram T, Neumark Y (2021). Community rehabilitation and hospitalizations among people with chronic psychotic disorder: is there a differential association by co-occurring substance use disorder?. Front Psychiatry.

[27] Florentin S, Neumark Y, Raskin S, Bdolah-Abram T, Rosca P (2021). Differential effect of community rehabilitation reform on hospitalizations of patients with chronic psychotic disorders with and without substance use disorder, Israel, 1991–2016. Adm Policy Ment Health.

[28] Florentin S, Rosca P, Raskin S, Bdolah-Abram T, Neumark Y (2019). Psychiatric Hospitalizations of Chronic psychotic disorder patients with and without dual diagnosis, Israel, 1963–2016. J Dual Diagn.

[29] Lichtenberg P, Kaplan Z, Grinshpoon A, Feldman D, Nahon D (1999). The goals and limitations of Israel’s psychiatric case register. Psychiatr Serv.

[30] Charlson FJ, Ferrari AJ, Santomauro DF (2018). Global epidemiology and burden of Schizophrenia: findings from the global burden of Disease Study 2016. Schizophr Bull.

[31] Boland R, Verduin M, Ruiz P. Kaplan & Sadock’s Synopsis of Psychiatry. 12th Edition. Philadelphia: Wolters Kluwer, 2021.

[32] Central Bureau of Statistics of Israel. The Moslem Population in Israel. 2021. Available at: https://www.cbs.gov.il/he/mediarelease/DocLib/2021/233/11_21_233e.pdf.

[33] Rosca P, Spivak P, Goldman K, Austin O. Department for the Treatment of Substance Abuse - Ministry of Health, Annual Report, 2019, pp. 33–34 (in Hebrew).

[34] Elroy I, Samuel H, Medina-Artom T, -JDC-Brookdale Institute Smokler Center for Health Policy Research. The Shortage of Arab Professionals in Mental-Health Services — Causes and Solutions. Myers. 2018 Available at: https://brookdale-web.s3.amazonaws.com/uploads/2018/05/Eng_Summary_767_18.pdf.

[35] Jorm AF. Public knowledge and awareness about mental illnesses. In: Thornicroft G, Szmukler G, Mueser KT, Drake RE, editors. Oxford Textbook of Community Mental Health. Oxford University Press; 2011. pp. 247–52.

[36] Dardas LA, Simmons LA (2015). The stigma of mental illness in arab families: a concept analysis. J Psychiatr Ment Health Nurs.

[37] Zolezzi M, Alamri M, Shaar S, Rainkie D (2018). Stigma associated with mental illness and its treatment in the arab culture: a systematic review. Int J Soc Psychiatry.

[38] Fahoum K, Al-Krenawi A. Perceptions of stigma toward mental illness in arab society in Israel. Int Social Work. 2021;0(0). 10.1177/00208728211018727.

[39] Baruch DE, Kanter JW, Pirutinsky S, Murphy J, Rosmarin DH. Depression stigma and treatment preferences among Orthodox and non-Orthodox Jews [published correction appears in J Nerv Ment Dis. 2014;202(11):839. Rosmain, David H [Corrected to Rosmarin, David H]]. J Nerv Ment Dis. 2014;202:556 – 61. 10.1097/NMD.0000000000000158. 10.1097/NMD.0000000000000158 24921418

[40] Greenberg D, Witztum E (2013). Challenges and conflicts in the delivery of mental health services to ultra-orthodox Jews. Asian J Psychiatr.

[41] Silverstein M, Lowenstein A, Katz R, Gans D, Fan YK, Oyama P (2013). Intergenerational support and the Emotional Well-being of older Jews and Arabs in Israel. J Marriage Fam.

[42] Peled K (2013). The israeli Arab extended family and the inner courtyard: a historical portrait. Isr Affairs.

[43] Jacobs R, Gutacker N, Mason A (2015). Determinants of hospital length of stay for people with serious mental illness in England and implications for payment systems: a regression analysis. BMC Health Serv Res.

[44] Cheng P, Wang L, Xu L, Zhou Y, Zhang L, Li W (2022). Factors related to the length of stay for patients with Schizophrenia: a retrospective study. Front Psychiatry.

[45] Chen S, Collins A, Anderson K, McKenzie K, Kidd S, Patient, Characteristics (2017). Length of Stay, and functional improvement for Schizophrenia Spectrum Disorders: a Population Study of Inpatient Care in Ontario 2005 to 2015. Can J Psychiatry.

[46] McHugh RK, Votaw VR, Sugarman DE, Greenfield SF (2018). Sex and gender differences in substance use disorders. Clin Psychol Rev.

[47] Vitman-Schorr A, Ayalon L (2020). The changing status of israeli Arab Women as reflected in their role as main caregivers. J Fam Issues.

[48] Meah L, Reflection, Cote M, Gilbert P (2007). The muslim community and mental health care. Nichols. Spirituality, values, and mental health: jewels for the journey.

[49] Vu M, Azmat A, Radejko T, Padela AI (2016). Predictors of delayed Healthcare seeking among american Muslim Women. J Womens Health (Larchmt).

[50] Saherwala Z, Bashir S, Gainer D (2021). Providing culturally competent Mental Health Care for Muslim Women. Innov Clin Neurosci.

[51] Mohammadi N, Evans D, Jones T (2007). Muslims in australian hospitals: the clash of cultures. Int J Nurs Pract.

[52] Douki S, Zineb SB, Nacef F, Halbreich U (2007). Women’s mental health in the muslim world: cultural, religious, and social issues. J Affect Disord.

[53] Giordano GM, Bucci P, Mucci A, Pezzella P, Galderisi S (2021). Gender differences in clinical and psychosocial features among persons with schizophrenia: a mini review. Front Psychiatry.

[54] Li X, Zhou W, Yi Z (2022). A glimpse of gender differences in schizophrenia. Gen Psychiatr.

[55] Selvendra A, Toh WL, Neill E (2022). Age of onset by sex in schizophrenia: proximal and distal characteristics. J Psychiatr Res.

[56] Zreik G, Golden D, Oppenheim D (2022). Challenges of Mothering in Extended families: the case of palestinian women in Israel. J Cross-Cult Psychol.

[57] Abu Ghanem S. Modernization in Israeli Arab Society – Parent Intervention Prog. Studia Edukacyjne nr 54, 2019, Poznań. 2019;335–343. Adam Mickiewicz University Press. ISSN 1233–6688. 10.14746/ se.2019.54.20.

